# Palliative care** – **too complex to make it simple

**DOI:** 10.3325/cmj.2024.65.165

**Published:** 2024-04

**Authors:** Aleksandar Džakula, Karmen Lončarek, Dorja Vočanec

**Affiliations:** 1 Department of Social Medicine and Organization of Health Care, Andrija Štampar School of Public Health, University of Zagreb School of Medicine, Zagreb, Croatia *dvocanec@snz.hr*; 2 Center for Integrated and Palliative Care, University of Rijeka, Faculty of Medicine, Rijeka, Croatia

You matter because you are you. You matter to the last moment of your life, and we will do all we can, not only to help you die peacefully, but also to live until you die.

Cicely Saunders, The Management of Terminal Disease (1)

*The old Mediterranean norm that a wise person needs to acquire and treasure an* amicus mortis, *one who tells you the bitter truth and stays with you to the inexorable end - calls for revival. And I see no compelling reason why one who practices medicine could not also be a friend – even today.*

Ivan Illich, Death Undefeated (2)

Every day, medicine becomes more and more complicated, and health care becomes more and more complex. Despite all the hopes and promises of modern science, death has not been defeated. We are as mortal as we were at the dawn of the human species, and will remain so for an infinite time. But what about dying? Has dying remained the same or does it too evolve into a more complex phenomenon?

The 20th century brought unparalleled progress in medicine with almost incredible possibilities of diagnosis and treatment. However, not only was death not conquered, but it became a taboo subject. Namely, death is contrary to the idea of healing that comes with new technologies and drugs, and dying is contrary to the promises of eternal youth that these technologies give us so wholeheartedly.

This ignoring of death and dying lasted until the 1960s and the emergence of the hospice movement in Great Britain. The revolution started by Cicely Saunders brought the concepts of death, dying, and suffering back into the focus of society and medicine. This is why the work of Cicely Saunders is considered one of the most significant achievements of modern health care (1).

Cicely Saunders and her contributions in the form of the hospice movement represent a fundamental shift in the way society and medicine approach death, dying, and suffering in the last few decades. Previously, modern medicine focused mainly on the treatment and eradication of disease, often neglecting patients’ quality of life in its terminal stages. Saunders, however, emphasized the importance of a holistic approach to the care of the dying, advocating not only the relief of physical suffering, but also emotional, social, and spiritual support as integral part of end-of-life care (3).

This accomplishment, although incredibly important, carries a paradoxical weight. Namely, it forces acknowledgment of the self-evident facts that people are mortal and that they suffer. This confrontation with mortality and suffering proved to be the greatest challenge of modern medicine. Another author who contributed significantly to the critical analysis of modern medicine and its impact on society was Ivan Illich. He was one of the first thinkers to express concern about the medicalization of life wherein medicine takes over aspects of the human experience beyond those traditionally considered medical issues, such as aging and dying, and even some aspects of everyday life. In “Medical Nemesis,” Ilich specifically pointed out that the process of dying, like many other aspects of the human experience, is becoming more complex – the way we approach, understand, and manage it has been shaped under the influence of social, technological, and medical progress (4).

Reducing suffering and creating conditions for a dignified end of life were key priorities of the hospice movement and palliative care. A whole series of new medical technologies has been developed to meet these goals. In addition to technologies and devices that add quantity to life, new technologies that increase the quality of life also play a significant role. There are various solutions for nutritional support, breathing support, aids that delay dependence when performing activities of daily living, and numerous excellent analgesics (5,6).

In other words, modern technologies have made the end of life easier, and have enabled patients and caregivers to significantly improve the quality of the end of life. At the same time, these technologies made that period much longer, and also much more complex.

The emergence of these new medical technologies not only posed a challenge for resource provision and organization, but also raised two new important perspectives. One is the need for legal regulation regarding their use in palliative care, and the other is ethical. Every new technology or organizational solution requires a legal basis, that is, a legal framework in which they can be implemented. Legal solutions can hardly keep up with the pace at which new technologies are created. On the other hand, ethical issues arise, both general and specific for the individual patient, their family, and their environment. Addressing these challenges requires a collaborative effort involving medical professionals, lawyers, ethicists, patients, and patients’ families. Developing guidelines, educational programs, and ethical frameworks that keep up with technological advances is essential to ensuring that new medical technology is used in the best interests of everyone involved.

Despite all the talk about how technology changes society, the role of society, community, and the immediate environment on the sick and dying is not highlighted enough. It appears as if the fascination with technology has overshadowed the vital role played by the network of individuals surrounding the seriously ill and dying. And it is within this network and relationships that you can see how well and comprehensively we care for patients and their complex end-of-life needs (7,8). Drawing on Ivan Illich’s analogy of “*amicus mortis*” from his article on unconquered death, it seems we must return to this ancient model. The concept involved a trusted individual who conveyed the bitter truth to the dying person while providing unwavering support until the end (2).

It appears that we have reached a time when each of us needs our own “*amicus mortis*,” someone who can compassionately deliver difficult news and guide us through the complexities of our world. How can we comprehend our circumstances and position ourselves amidst the complicated and complex reality that surrounds us? Perhaps now more than ever, we need that new “*amicus mortis*” equipped to confront the complex world that surrounds us.
